# Conjugation of *Glycine max* (L.) Merrill Oligopeptide with Monosaccharides: A Novel Approach for Stability and Efficacy in Cosmeceutical Applications

**DOI:** 10.3390/pharmaceutics17040530

**Published:** 2025-04-17

**Authors:** Wantida Chaiyana, Sudarat Jiamphun, Rewat Phongphisutthinant, Supakit Chaipoot, Pairote Wiriyacharee

**Affiliations:** 1 Department of Pharmaceutical Sciences, Faculty of Pharmacy, Chiang Mai University, Chiang Mai 50200, Thailand; 2 Multidisciplinary and Interdisciplinary School, Chiang Mai University, Chiang Mai 50200, Thailand; 3 Center of Excellence in Pharmaceutical Nanotechnology, Faculty of Pharmacy, Chiang Mai University, Chiang Mai 50200, Thailand; 4 School of Cosmetic Science, Mae Fah Luang University, Chiang Rai 57100, Thailand; sudarat.jia@mfu.ac.th; 5 Multidisciplinary Research Institute, Chiang Mai University, Chiang Mai 50200, Thailand; rewat.p@cmu.ac.th (R.P.); supakit.ch@cmu.ac.th (S.C.); 6 Research Center of Microbial Diversity and Sustainable Utilization, Faculty of Science, Chiang Mai University, Chiang Mai 50200, Thailand; pairote.w@cmu.ac.th; 7 Faculty of Agro-Industry, Chiang Mai University, Chiang Mai 50100, Thailand

**Keywords:** conjugate, mannose, allulose, soybean, biological stability, acid–base precipitation, cosmeceuticals, antioxidant, whitening, anti-wrinkles

## Abstract

**Background/Objectives**: Conjugation techniques are increasingly valued in food chemistry for enhancing sensory properties, nutritional profiles, and bioactivity, with potential applications in cosmeceuticals. This study aimed to investigate the potential of *Glycine max* (L.) Merrill oligopeptide–monosaccharide conjugates as active ingredients in cosmeceuticals, emphasizing their biological activities and stability. **Methods**: *G. max* isolate was prepared and subsequently hydrolyzed using alcalase to obtain the oligopeptide (OP). The OP was then conjugated with allulose (AL) or mannose (MN) through a controlled humid-dry heating process to produce the conjugates, OPA and OPM, respectively. Their biological activities, including antioxidant, anti-tyrosinase, anti-collagenase, anti-elastase, and anti-hyaluronidase properties, were assessed and compared to the individual components. Additionally, the irritation potential was evaluated using the hen’s egg test on chorioallantoic membrane (HET-CAM). The stability was examined under varying pH levels, temperatures, and light conditions based on their biological activity profiles. **Results**: OPA demonstrated the highest antioxidant activity, showing the lowest DPPH^•^ IC_50_ value of 198.6 ± 2.7 µg/mL along with a strong ferric reducing power of 1.37 ± 0.04 µg FeSO_4_/g sample. Besides, OPM showed superior tyrosinase inhibition on both L-tyrosine and L-DOPA substrates, highlighting its potential for skin whitening. Both OPA and OPM significantly enhanced collagenase inhibition, supporting their anti-aging potential. All samples were non-irritating in the HET-CAM test. The conjugates (OPA and OPM) demonstrated enhanced stability against pH, heat, and light compared to OP, AL, and MN. **Conclusions**: Oligopeptide–monosaccharide conjugation not only improved bioactivity but also enhanced biological stability, suggesting their potential for use in cosmeceutical applications.

## 1. Introduction

*Glycine max* (L.) Merrill, commonly known as soybean, is a widely consumed species due to its rich nutritional profile and bioactive properties. *G. max* has a protein content of 40%, making it superior to most other beans, and provides high-quality protein comparable to dairy, meat, and eggs, but without saturated fats and cholesterol [1]. Aside from their high protein content and superior protein quality, *G. max* contains rich levels of secondary metabolites such as isoflavones, saponins, phytic acids, phytosterols, trypsin inhibitors, peptides, and essential amino acids [2,3]. Besides its nutritional and nutraceutical values, *G. max* has been used in the cosmetic industry. *G. max* proteins and peptides primarily served as effective skin and hair conditioning agents in personal care formulations [4]. *G. max* extracts have demonstrated the ability to inhibit elastases while promoting the production of elastin, collagen, and glycosaminoglycans, especially hyaluronic acid, in aging skin [5,6,7]. Soymilk and its derived proteins have been reported to promote skin depigmentation by inhibiting the phagocytosis of melanosomes by keratinocytes, thereby reducing the transfer of melanin [6]. Aside from their depigmenting efficacy, soymilk proteins have also demonstrated the ability to inhibit UV-induced pigmentation [8]. Therefore, *G. max*, particularly its proteins and hydrolyzed derivatives, has demonstrated a range of bioactivities related to cosmetic applications, including skin conditioning, anti-aging, and whitening effects [4].

One of the challenges in using *G. max* in cosmetic formulations is that its raw extracts, such as proteins and peptides, can be sensitive to environmental factors. Proper protein folding is crucial for its biological function, as the specific three-dimensional structure determines its activity and interaction with other molecules [9]. However, protein stability is highly sensitive to environmental factors, which can cause denaturation or unfolding, leading to a loss of function and effectiveness. Additionally, the natural odor of *G. max* extracts can sometimes be undesirable in cosmetic products. To address these issues, conjugating *G. max* compounds with other molecules would provide a solution by improving their stability and bioactivity. Although conjugation techniques have gained attention in food chemistry for enhancing the stability, sensory properties, and nutritional value of bioactive compounds, their potential applications in cosmetics are also promising [10,11].

Proteins and saccharides are two fundamental classes of bioactive substances, and recent research has focused on their covalent interactions to improve functional properties and bioactivity [12]. Conjugating proteins with saccharides forms glycoproteins, where an oligosaccharide chain is covalently bonded to specific amino acid residues, typically through the Maillard reaction, which links protein amino residues (NH_2_) with the reducing carbonyl groups (C=O) of saccharides, thereby altering the protein’s structure to enhance its functionality [13]. These conjugates have been reported for a range of bioactivities, including antioxidant, antibacterial, immunoregulatory, and anticancer effects [14]. Various natural glycoproteins have been reported for their beneficial effects in the cosmetic industry, including those found in silk [15], milk [16], rose [17], carrot [18], etc. Therefore, *G. max* oligopeptides, which have already been reported to possess various cosmeceutical properties, could exhibit even greater potential following conjugation, enhancing their bioactivity and stability for more effective applications in cosmetic formulations. Additionally, conjugation may improve the stability and bioavailability of active ingredients, making them more resilient to environmental factors such as temperature, pH, and light exposure.

Therefore, this study aimed to investigate the potential of *G. max* oligopeptide–monosaccharide conjugates as active ingredients in cosmeceuticals, with a focus on their biological activities and stability. Their bioactive properties, including antioxidant, skin-whitening, and anti-aging effects, along with their irritation potential, were also assessed. Furthermore, the stability of both the *G. max* oligopeptide–monosaccharide conjugates and their individual components was evaluated under varying environmental conditions to ensure their efficacy in cosmeceutical applications.

## 2. Materials and Methods

### 2.1. Chemical Materials

*G. max* oligopeptide was obtained from the Faculty of Agro-Industry, Chiang Mai University, Chiang Mai, Thailand. Alcalase enzyme (EC 3.4.21.14), allulose (AL), ascorbic acid, collagenase from *Clostridium histolyticum* (EC 3.4.23.3), epigallocatechin gallate (EGCG), N-[3-(2-furyl) acryloyl]-Leu-Gly-Pro-Ala (FALGPA), hydrochloric acid (HCl), kojic acid, L-3,4 dihydroxyphenylalanine (L-DOPA), L-tyrosine, mannose (MN), oleanolic acid, porcine pancreatic elastase (EC 3.4.21.36), sodium chloride (NaCl) sodium hydroxide (NaOH), sodium lauryl sulfate (SLS), and tyrosinase from mushroom (EC 1.14.18.1) were analytical grades purchased from Sigma-Aldrich (St. Louis, MO, USA).

### 2.2. Production Process of G. max Oligopeptide

The production process of *G. max* oligopeptide began by isolating *G. max* proteins, which were then subjected to enzymatic hydrolysis to break them down into oligopeptides. To generate the *G. max* isolate, *G. max* powder (20 g) was dispersed in DI water (100 mL) and adjusted to the pH of 8–9 using 5 M NaOH solution. The resulting mixture was shaken at room temperature for 1 h and then centrifuged at 7000 rpm for 5 min. The supernatant was collected and adjusted to the pH of 5.0 using 5 M HCl. Following this, the resulting mixture was centrifuged at 7000 rpm for 5 min. After the removal of the supernatant, the precipitate was then desalted using 10 kDa dialysis tubing for 72 h, freeze-dried, and the fat was extracted using hexane to obtain the *G. max* isolate. Subsequently, the *G. max* isolate powder (5 g) was dispersed in DI water (200 mL). Following this, 1 mL of Alcalase enzyme solution was added. The mixture was shaken at 50 °C for 5 h, followed by a reaction stop at 95 °C for 15 min. After cooling to room temperature, the mixture was filtered through Whatman No. 1 filter paper. The filtrate was then collected and freeze-dried to obtain the *G. max* oligopeptide (OP). The total protein content of OP, evaluated through a Dumas combustion assay to determine the nitrogen content and converted to total protein using a conversion factor of 6.25, was 58.31 g/100 g [19]. Additionally, five predominant amino acids were detected through high-performance liquid chromatography (Shimadzu, Kyoto, Japan) using a sodium-type cation-exchange column (100 mm × 6.0 mm ID, 5 μm; P/N: 228-18837-91, Shimadzu, Japan), including proline (44.62 mg/100 g), lysine (21.74 mg/100 g), phenylalanine (11.27 mg/100 g), histidine (8.89 mg/100 g), and tyrosine (6.70 mg/100 g) [19]. The other amino acids were detected in trace amounts (0.15–1.71 mg/100 g), including aspartic acid, threonine, serine, glutamic acid, glycine, alanine, cysteine, valine, methionine, isoleucine, and arginine [19].

### 2.3. Production Process of G. max Oligopeptide–Monosaccharide Conjugates

Two types of monosaccharides, including allulose (AL) and mannose (MN), were used to produce *G. max* oligopeptide–monosaccharide conjugates in the current study. In brief, OP was dissolved in DI water at a 1:10 ratio. The protein concentration was adjusted to 1% *w*/*w*, based on the calculation that 100 mL of OP contains 1.28 g of protein. The OP was then mixed with 1% *w*/*v* AL or MN in a 1:1 volume ratio. The mixture was then freeze-dried and incubated at 60 °C with 80% relative humidity for 10 days. The *G. max* oligopeptide–allulose (OPA) and *G. max* oligopeptide–mannose (OPM) were obtained. The confirmation of the conjugation between oligopeptides and monosaccharides was carried out through two complementary approaches, including the evaluation of the degree of glycation using the ortho-phthalaldehyde (OPA) assay and molecular weight determination via sodium dodecyl sulfate–polyacrylamide gel electrophoresis (SDS-PAGE) [19,20]. The OPA assay quantitatively measured the depletion of free amino groups, thereby indicating the extent of glycation via the Maillard reaction [19]. In contrast, SDS-PAGE was used to analyze changes in molecular weight, where an upward shift in the peptide bands served as evidence of successful conjugation, suggesting the formation of higher molecular weight glycopeptide complexes [20].

### 2.4. Determination of Biological Activities Related to Cosmetic/Cosmeceutical Applications

#### 2.4.1. Antioxidant Activities

- 2,2-Diphenyl-1-Picrylhydrazyl (DPPH) Assay

The DPPH radical scavenging activity of the *G. max* oligopeptide–monosaccharide conjugates, along with their individual components (oligopeptide and monosaccharide), was assessed following the method of Chaiyana et al. (2023) [21]. In brief, 20 µL of the sample solution was introduced to 180 µL of 167 μM DPPH^•^ methanolic solution and at ambient temperature for 30 min, after which absorbance was recorded at 520 nm using a microplate reader (CLARIOstar PLUS, BMG Labtech, Ortenberg, Germany). The DPPH^•^ inhibition was calculated as:

DPPH^•^ inhibition (%) = [(C − S)/C] × 100, (1)

where C represents the absorbance of the control (without the sample), and S represents the absorbance with the sample. L-ascorbic acid was used as a positive control. All measurements were repeated in triplicate.

- Ferric Reducing Antioxidant Power (FRAP) Assay

The reducing capacity of the *G. max* oligopeptide–monosaccharide conjugates, along with their individual components (oligopeptide and monosaccharides), was assessed following the method of Chaiyana et al. (2023) [21]. In brief, 20 µL of the sample solution was introduced to 180 µL of FRAP reagent, which was freshly prepared by combining 10 mM TPTZ in 40 mM HCl, 20 mM FeCl_3_; aqueous solution, and 300 mM acetate buffer (pH 3.6) in a 1:1:10 ratio. After incubating at ambient temperature for 10 min, the absorbance was recorded at 595 nm using a microplate reader (CLARIOstar PLUS, BMG Labtech, Ortenberg, Germany). Ferrous sulfate (FeSO_4_) was used as a standard compound to generate a standard curve. The results were expressed as EC_1_, representing milligrams of FeSO_4_ equivalents per gram of the sample. L-ascorbic acid was used as a positive control. All measurements were repeated in triplicate.

#### 2.4.2. Anti-Tyrosinase Activities

The anti-tyrosinase activity of the *G. max* oligopeptide–monosaccharide conjugates, along with their individual components (oligopeptide and monosaccharides), was assessed following the method of Chaiyana et al. (2023) [21]. In brief, 20 µL of the sample solution was introduced to 80 μL of 125 units/mL tyrosinase enzyme solution in 50 mM PBS pH 6.8 and incubated at room temperature for 10 min. Following this, 100 μL of L-Tyrosin or L-DOPA in PBS pH 6.8 was introduced and at ambient temperature for 30 min, after which absorbance was recorded at 492 nm using a microplate reader (CLARIOstar PLUS, BMG Labtech, Ortenberg, Germany). The tyrosinase inhibition was calculated as:

tyrosinase inhibition (%) = [(C − S)/C] × 100,(2)

where C represents the absorbance of the control (without the sample), and S represents the absorbance with the sample. Kojic acid was used as a positive control. All measurements were repeated in triplicate.

#### 2.4.3. Anti-Skin Aging Activities

- Anti-Collagenase Activity

The collagenase inhibitory activity of the *G. max* oligopeptide–monosaccharide conjugates, along with their individual components (oligopeptide and monosaccharides), was assessed following the method of Thring et al. (2009) and Chaiyana et al. (2023) [21,22]. In brief, 20 µL of the sample solution was introduced to 80 μL of 5 units/mL collagenase solution in 50 mM Tricin buffer pH 7.4 and incubated at ambient temperature for 15 min. Following this, 100 μL of 2 mM FALGPA in 50 mM Tricin buffer pH 7.4 was introduced and at ambient temperature, after which absorbance was recorded at 492 nm continuously for 20 min using a microplate reader (CLARIOstar PLUS, BMG Labtech, Ortenberg, Germany). The collagenase inhibition was calculated as:

collagenase inhibition (%) = [(C − S)/C] × 100,(3)

where C represents the reaction rate, which was the slope of the plot of absorbance and the sample concentration of the control (without the sample), and S represents the reaction rate with the sample. EGCG was used as a positive control. All measurements were repeated in triplicate.

- Anti-Elastase Activity

The elastase inhibitory activity of the *G. max* oligopeptide–monosaccharide conjugates, along with their individual components (oligopeptide and monosaccharides), was assessed following the method of Thring et al. (2009) and Chaiyana et al. (2023) [21,22]. In brief, 20 µL of the sample solution was introduced to 80 μL of 0.042 units/mL elastase solution in 200 mM Tris-HCl buffer pH 8.0 and incubated at ambient temperature for 15 min. Following this, 100 μL of 1.6 mM AAAPVN in Tris-HCl buffer pH 8.0 was introduced and at ambient temperature, after which absorbance was recorded at 492 nm continuously for 20 min using a microplate reader (CLARIOstar PLUS, BMG Labtech, Ortenberg, Germany). The elastase inhibition was calculated as:

elastase inhibition (%) = [(C − S)/C] × 100,(4)

where C represents the reaction rate, which is the slope of the plot of absorbance and the sample concentration of the control (without the sample), and S represents the reaction rate with the sample. EGCG was used as a positive control. All measurements were repeated in triplicate.

- Anti-Hyaluronidase Activity

The hyaluronidase inhibitory activity of the *G. max* oligopeptide–monosaccharide conjugates, along with their individual components (oligopeptide and monosaccharides), was assessed following the method of Thring et al. (2009) and Chaiyana et al. (2023) [21,22]. In brief, 20 µL of the sample solution was introduced to 80 μL of 15 units/mL hyaluronidase solution in 20 mM PBS pH 7.0 containing 77 mM sodium chloride and 0.01% *w*/*v* BSA and incubated at 37 °C in the dark for 10 min. Following this, 100 μL of 300 mM hyaluronic acid solution in PBS pH 5.35 was introduced and incubated at 37 °C for 45 min. After mixing with 1 mL of 0.1 mg/mL acidic BSA solution in 24 mM acetate buffer pH 3.75, the absorbance was recorded at 600 nm using a microplate reader (CLARIOstar PLUS, BMG Labtech, Ortenberg, Germany). The hyaluronidase inhibition was calculated as:

elastase inhibition (%) = [(C − S)/C] × 100,(5)

where C represents the absorbance of the control (without the sample) and S represents the absorbance with the sample. Oleanolic acid was used as a positive control. All measurements were repeated in triplicate.

### 2.5. Irritation Test by Hen’s Egg Chorioallantoic Membrane (HET-CAM)

The irritation potential of the *G. max* oligopeptide–monosaccharide conjugates, along with their individual components (oligopeptide and monosaccharides), was evaluated using the HET-CAM assay, following the method of Chaiyana et al. (2023) [21]. This in vitro assay assessed skin irritation using the chorioallantoic membrane (CAM) of a chicken embryo aged 7–9 days, which provided a vascular network. However, ethical approval was waived, as the embryos in the early stages of development are not considered animals [23]. Typically, hen eggs require 21 days of incubation, and the fertilized eggs, aged between 7 and 9 days, are less than halfway through their incubation cycle and, therefore, do not fall under the category of animals according to ethical guidelines [24]. Additionally, the chicken embryo was not capable of experiencing pain until day 14 of incubation, as the development of its nervous system was not sufficiently advanced prior to this stage [25,26]. Fertilized hen eggs, aged 7–9 days, were obtained from the Faculty of Agriculture at Chiang Mai University, Thailand, and incubated at 37.5 ± 0.5 °C with 62.5 ± 7.5% humidity. The eggshell above the air cavity was removed using a rotating blade, and the inner membrane was exposed to normal saline solution (NSS) and incubated for 15 min. The sample solution in NSS (30 µL at 10 mg/mL) was then applied to the CAM, with positive and negative controls being 1% *w*/*v* sodium lauryl sulfate (SLS) and NSS, respectively. Irritation effects were observed under a stereomicroscope over 5 min and 60 min. Signs of irritation, including vascular hemorrhage, lysis, and coagulation, were observed, and the time in seconds of their first observation was used for the calculation of the irritation score (IS) using a specific equation as follows:

IS = 5(1 − H/300) + 7(1 − L/300) + 9(1 − C/300)(6)

where A represents the times of initial appearance of specific signs, including H for vascular hemorrhage, L for vascular lysis, and C for vascular coagulation. The irritation severity was categorized as follows: IS = 0.0 to 0.9 (no irritation), IS = 1.0 to 4.9 (mild irritation), IS = 5.0 to 8.9 (moderate irritation), and IS = 9.0 to 21.0 (severe irritation).

### 2.6. Stability Test

*G. max* oligopeptide–monosaccharide conjugates, along with their individual components (oligopeptide and monosaccharides), were evaluated for their stability under various conditions for up to four weeks. Factors affecting their stability were assessed, including pH levels (3, 5, 7, and 9), temperatures (4 °C, 25 °C, and 45 °C), and light exposure. The samples were evaluated for their biological activities, including ferric-reducing ability, anti-tyrosinase, and anti-collagenase activities, as described above, after storage for 1, 2, and 4 weeks. The results were reported as the remaining biological activities, which were calculated using a specific equation as follows:

remaining biological activities = (A − B) × 100,(7)

where A represents the biological activities after the specific storage period under each condition and B represents the biological activities before storage. All measurements were repeated in triplicate.

### 2.7. Statistical Analysis

All measurements were conducted in triplicate, and the results are presented as mean ± S.D. Statistical analysis was performed using SPSS Software, Version 17.0 for Windows. Differences between groups were assessed using one-way analysis of variance (ANOVA) followed by Tukey’s test, with significance set at a *p*-value ≤ 0.05.

## 3. Results and Discussion

### 3.1. G. max Oligopeptide–Monosaccharide Conjugates

**Figure 1:**
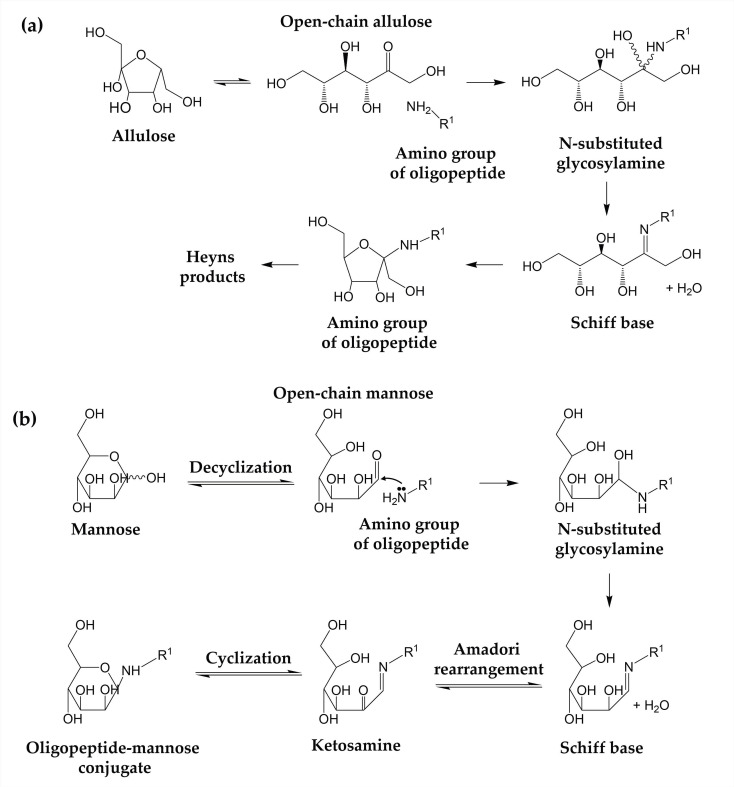
Schematic representation of Maillard reaction pathways between oligopeptides and the reducing sugars: (**a**) allulose; (**b**) mannose.

Conjugation between *G. max* oligopeptides and monosaccharides was successfully achieved, as confirmed by two complementary analytical approaches. The reduction in free amino groups, measured by the OPA assay, indicated the extent of glycation, while SDS-PAGE analysis revealed an increase in the molecular weight of the peptides, supporting the formation of glycopeptide conjugates [19,20]. The Maillard reaction begins with the interaction between reducing sugars and suitable amines (such as amino acids, peptides, or proteins), involving two well-known non-enzymatic rearrangements, including the Amadori rearrangement for aldose sugars and the Heyns rearrangement for ketose sugars [27]. In the current study, two types of monosaccharides were used for conjugation with *G. max* oligopeptides, including allulose, a ketose sugar and C-3 epimer of fructose, and mannose, an aldose sugar and C-2 epimer of glucose. Figure 1 presents the proposed Maillard-type reaction pathways between the reducing sugars (allulose and mannose) and the amino groups of oligopeptides.

Allulose (Figure 1a), a ketose sugar, undergoes ring opening to yield an open-chain form, which reacts with the amino group of an oligopeptide to form an N-substituted glycosylamine. This intermediate may undergo dehydration to produce a Schiff base, which can further rearrange into Heyns products [28]. This pathway is characteristic of ketose sugars like allulose, often resulting in structurally diverse rearranged products that may influence the biochemical properties of the conjugates [29]. Among different ketose sugars, allulose has been reported to have higher rates of Maillard reaction than fructose [29,30]. On the other hand, Figure 1b illustrates a comparable pathway for mannose. Upon decyclization, the open-chain mannose reacts with the amino group of oligopeptide to form a glycosylamine, followed by the formation of a Schiff base [31]. This intermediate can undergo Amadori rearrangement, yielding a stable ketosamine, which may subsequently cyclize into a stable oligopeptide–mannose conjugate [32].

### 3.2. Antioxidant Activities of G. max Oligopeptide–Monosaccharide Conjugates

**Figure 2:**
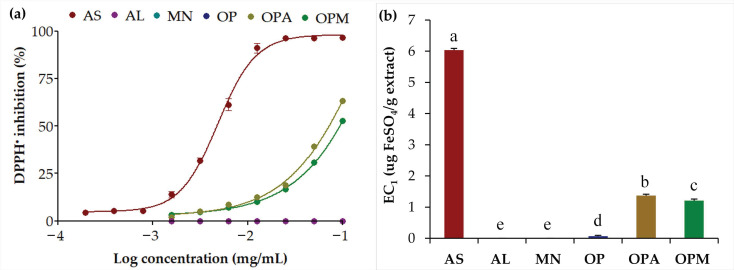
Antioxidant activities: (**a**) dose-response curve for DPPH^•^ inhibition and (**b**) equivalent concentration (EC_1_) for ferric-reducing antioxidant power of ascorbic acid (AS; maroon), allulose (AL; purple), mannose (MN; cyan), *G. max* oligopeptide (OP; dark blue), *G. max* oligopeptide–allulose (OPA; yellow), and *G. max* oligopeptide–mannose (OPM; green). Different letters (a, b, c, d, and e) denote significant differences in the antioxidant activities among samples, as determined by ANOVA followed by Tukey’s test (*p* < 0.05).

To assess the antioxidant activity, two methods based on different mechanisms were used in the current study. The DPPH assay is widely used to assess the ability to donate hydrogen atoms or electrons to neutralize free radicals, while the FRAP assay measures the reducing power of an antioxidant by its ability to convert ferric (Fe^3+^) to ferrous (Fe^2+^) ions [33]. The antioxidant activities of *G. max* oligopeptide–monosaccharide conjugates are shown in Figure 2. No DPPH inhibition was observed in the individual compounds (AL, MN, and OP), as shown in Figure 2a, as their graphical plots completely overlapped with the 0% inhibition line. Similarly, the individual compounds demonstrated either an absence of reducing power or only a negligible level of activity, with equivalent concentrations approaching zero, as shown in Figure 2b. While the individual monosaccharides, both AL and MN, along with OP, exhibited negligible antioxidant activities on their own, their conjugation resulted in significant antioxidant properties. The enhancements were evident through both the DPPH radical scavenging activities and ferric-reducing antioxidant power. The likely explanation could be the Maillard reaction between oligopeptides and monosaccharides, which occurred when the amino group (-NH_2_) in an oligopeptide reacted with the carbonyl group (-C=O) in monosaccharides, resulting in glycation compounds with new structures exhibiting enhanced antioxidant activity and improved functional properties [34]. The findings were in line with a previous study that reported peptides could undergo the Maillard reaction, easily cross-linking with sugars to form compounds with good antioxidant activity [35]. In addition, the Maillard reaction products formed from amino acid-sugar reactions exhibit strong antioxidant activity through various mechanisms, including radical chain-breaking activity, scavenging of reactive oxygen species, decomposition of hydrogen peroxide, and metal chelation [34].

AL and MN are both hexose monosaccharides but differ in their structural composition and functional groups. While AL is a ketohexose with a ketone at C-2, forming a five-membered furanose ring, mannose is an aldohexose with an aldehyde at C-1, forming a six-membered pyranose ring [36]. The conjugation of both monosaccharides with OP resulted in different antioxidant activities. OPA inhibited the DPPH^•^ radical with an IC_50_ value of 198.6 ± 2.7 µg/mL, while OPM exhibited an IC_50_ value of 374.4 ± 0.8 µg/mL, suggesting that OPA is more potent. The results were in line with the ferric reducing potential, revealing a higher EC_1_ value for OPA (1.37 ± 0.04 µg FeSO_4_/g sample) compared to OPM (1.21 ± 0.05 µg FeSO_4_/g sample). The *G. max* oligopeptide–monosaccharide conjugates, despite not exhibiting antioxidant potency equivalent to ascorbic acid, demonstrated promising antioxidant properties that can benefit skin health. As antioxidants inhibit oxidation by preventing oxidation-reduction reactions that generate free radicals, which can trigger harmful chain reactions [37], they play a vital role in protecting against cellular damage. Residual free radicals in the body contribute to aging by damaging healthy skin and tissue cells, while antioxidants help mitigate these effects and support cellular health [37,38]. Therefore, the *G. max* oligopeptide–monosaccharide conjugates, particularly OPA, were attractive for use in the cosmetic/cosmeceutical area.

### 3.3. Anti-Tyrosinase Activities of G. max Oligopeptide–Monosaccharide Conjugates

**Figure 3:**
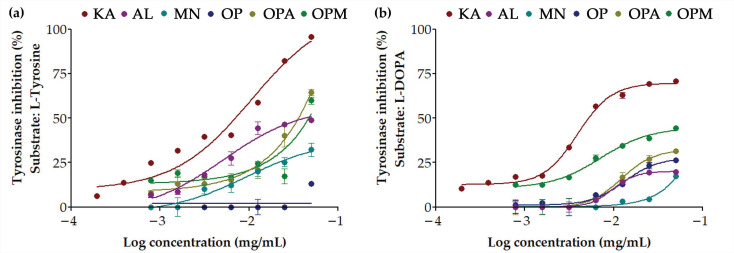
Dose-response curve for anti-tyrosinase activities against different substrates: (**a**) L-tyrosine and (**b**) L-DOPA of kojic acid (KA; maroon), allulose (AL; purple), mannose (MN; cyan), *G. max* oligopeptide (OP; dark blue), *G. max* oligopeptide-allulose (OPA; yellow), and *G. max* oligopeptide-mannose (OPM; green).

Tyrosinase is an enzyme that plays a pivotal role in the biosynthesis of melanin, the pigment responsible for skin color [39,40]. Tyrosinase exhibited two distinct activities, including monophenolase (cresolase) activity through hydroxylated L-tyrosine to L-DOPA and diphenolase (catecholase) activity through oxidizing L-DOPA to dopaquinone [39]. The inhibition of tyrosinase, hence, reduced melanin synthesis, leading to skin lightening or whitening effects. This approach is commonly used in treating hyperpigmentation disorders and in cosmetic products aimed at achieving a more even skin tone [41]. The anti-tyrosinase activities of *G. max* oligopeptide–monosaccharide conjugates have been observed to be dose-dependent, as shown in Figure 3. OP alone exhibited almost no inhibitory activity when L-tyrosine was the substrate but showed higher inhibitory activity with L-DOPA (Table 1). Conversely, both monosaccharides were more potent inhibitors when L-tyrosine was the substrate and less effective with L-DOPA. MN was found to exhibit lower anti-tyrosinase activity for both L-tyrosine and L-DOPA compared to AL. Interestingly, OPM displayed improved inhibitory activity compared to monosaccharides or OP alone for both L-tyrosine and L-DOPA, indicating that conjugation with MN enhanced its anti-tyrosinase activity. In contrast, OPA exhibited a different trend, showing enhancement only in the case of L-DOPA but with lower inhibition compared to OPM. Therefore, OPM appears to be the most promising as it inhibited tyrosinase at both stages, while OPA may be useful in formulations targeting later-stage melanin synthesis.

### 3.4. Anti-Skin Aging Activities of G. max Oligopeptide–Monosaccharide Conjugates

Skin deterioration with age is an inevitable process that involves a reduction in collagenous and elastic fibers [42,43]. As collagen is a major structural component of the skin, its degradation leads to the formation of wrinkles [44]. Collagen is known to be degraded by collagenase, and long-term elevations in the collagenase likely result in disorganized and clumped collagen in the skin, leading to a weakening of the skin’s structural integrity [45,46]. Aside from collagen, elastin is a crucial protein that imparts elasticity and resilience to the skin [47]. The increased elastase activity has been linked to premature skin aging, manifesting as wrinkles and reduced skin elasticity [48]. On the other hand, hyaluronic acid is an extracellular matrix component known for its exceptional ability to bind and retain water, contributing to skin hydration and elasticity [49]. Therefore, hyaluronic acid is a key ingredient in many skincare products aimed at enhancing skin hydration and combating signs of aging. Another approach to inhibit the degradation of hyaluronic acid by the enzyme hyaluronidase would help maintain skin hydration and elasticity [50].

**Figure 4:**
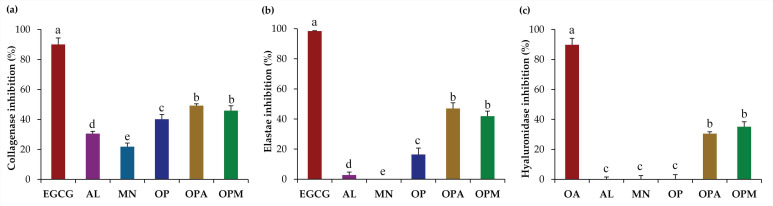
Anti-skin aging activities: (**a**) collagenase inhibition; (**b**) elastase inhibition; (**c**) hyaluronidase inhibition of epigallocatechin-3-gallate (EGCG; maroon), oleanolic acid (OA; maroon), allulose (AL; purple), mannose (MN; cyan), *G. max* oligopeptide (OP; dark blue), *G. max* oligopeptide–allulose (OPA; yellow), and *G. max* oligopeptide–mannose (OPM; green). The final concentration of the tested sample was 0.5 mg/mL. Different letters (a, b, c, d, and e) denote significant differences in the anti-skin aging activities among samples, as determined by ANOVA followed by Tukey’s test (*p* < 0.05).

The anti-skin aging activities of *G. max* oligopeptide–monosaccharide conjugates through the inhibitory activities against collagenase, elastase, and hyaluronidase, are shown in Figure 4. It was noted that the *G. max* oligopeptide–monosaccharide conjugates, both OPA and OPM, showed significantly improved anti-skin aging activities compared to the oligopeptide or monosaccharide alone. The collagenase inhibitory activities of OPA and OPM were significantly enhanced, reaching 49.1 ± 1.2% and 45.8 ± 3.3%, respectively, compared to the lower inhibitory activities of 30.5 ± 1.5%, 21.8 ± 2.5%, and 40.1 ± 3.1% for AL, MN, and OP, respectively. On the other hand, the inhibitory effects against elastase and hyaluronidase were significantly more pronounced. The monosaccharides, both AL and MN, exhibited no inhibitory effect on elastase and hyaluronidase; however, their conjugation with OP resulted in a remarkable enhancement of inhibitory effects. The current study suggested that the conjugation of *G. max* oligopeptides with monosaccharides significantly enhanced their inhibitory effects on collagenase, elastase, and hyaluronidase, highlighting their potential as anti-aging agents in skincare formulations.

### 3.5. Irritation Profile of G. max Oligopeptide–Monosaccharide Conjugates

**Figure 5:**
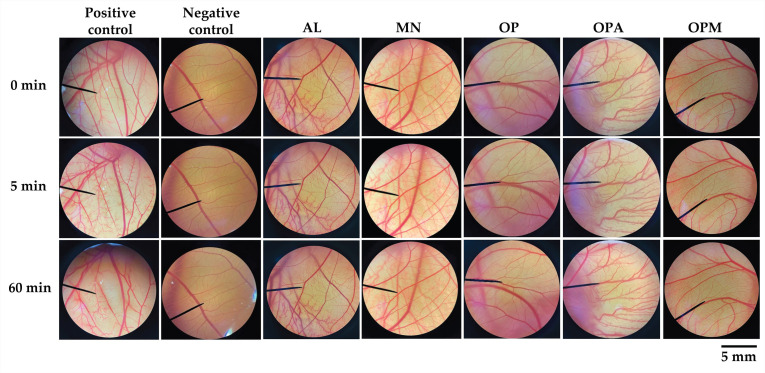
The photographs of the chorioallantoic membrane (CAM) at 0 min (before exposure) and after 5 and 60 min of exposure to the tested compounds, under a stereomicroscope with a 10× objective magnification. Sodium lauryl sulfate (SLS) at 1% *w*/*v* served as a positive control, while normal saline solution (NSS) served as a negative control. The tested sample of allulose (AL), mannose (MN), *G. max* oligopeptide (OP), *G. max* oligopeptide–allulose (OPA), and *G. max* oligopeptide–mannose (OPM) were tested at the concentration of 1% *w*/*w*.

The HET-CAM assay was performed to assess the irritation potential of the *G. max* oligopeptide–monosaccharide conjugates. The CAM exposed to the tested samples are shown in Figure 5, and their irritation score is listed in Table 2. SLS at 1% *w*/*w*, a well-known irritant, caused severe irritation, with an IS of 12.5 ± 0.3, while NSS showed no irritation. Exposure to SLS led to rapid irritation, including hemorrhage, coagulation, and vascular lysis, which became more pronounced over time. In contrast, *G. max* oligopeptide–monosaccharide conjugates, along with their individual oligopeptide and monosaccharide components, were tested, and no signs of irritation were observed after 5 or 60 min. The findings confirmed the reliability of the HET-CAM assay in predicting irritation and its suitability for assessing the safety of cosmetic ingredients. The results correlated well with human skin irritation tests, demonstrating its effectiveness as a preclinical screening tool [51]. This assay provided an initial evaluation before clinical studies, reducing the need for animal testing [52,53]. The absence of irritation signs in the *G. max* oligopeptide–monosaccharide conjugates suggested their safety for further applications in the cosmetic/cosmeceutical area.

### 3.6. Stability of G. max Oligopeptide–Monosaccharide Conjugates

Various factors affecting the stability of *G. max* oligopeptide–monosaccharide conjugates and their individual components were evaluated, including pH, temperature, and light exposure. The promising cosmeceutical properties assessed included ferric-reducing ability, anti-tyrosinase activities (on both L-tyrosine and L-DOPA), and anti-collagenase activities. The inhibitory activities against DPPH^•^ radicals, elastase, and hyaluronidase were excluded from the stability test because some compounds exhibited low or undetectable activities, making it impossible to observe changes in these biological activities during storage under various conditions.

**Figure 6:**
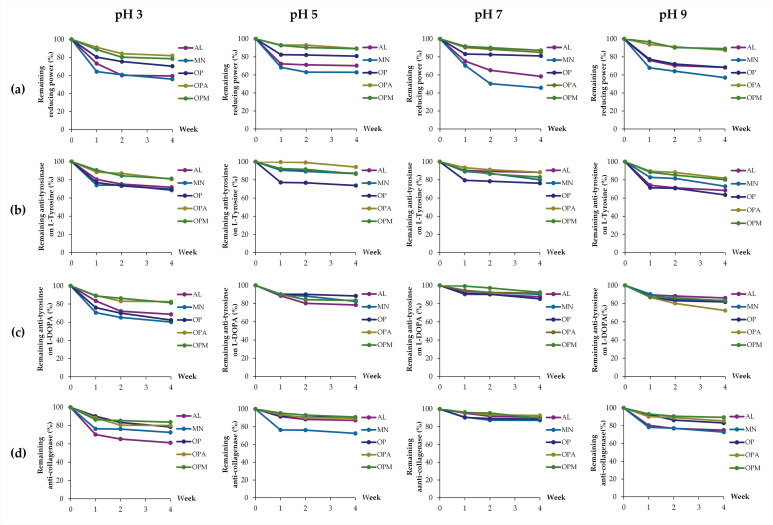
Effects of pH on stability, expressed as remaining biological activities: (**a**) ferric-reducing ability; (**b**) anti-tyrosinase activities on L-tyrosine; (**c**) anti-tyrosinase activities on L-DOPA; (**d**) anti-collagenase activities of allulose (AL; purple), mannose (MN; cyan), *G. max* oligopeptide (OP; dark blue), *G. max* oligopeptide-allulose (OPA; yellow), and *G. max* oligopeptide-mannose (OPM; green) after storage in various pH of 3, 5, 7, and 9 for up to 4 weeks.

**Figure 7:**
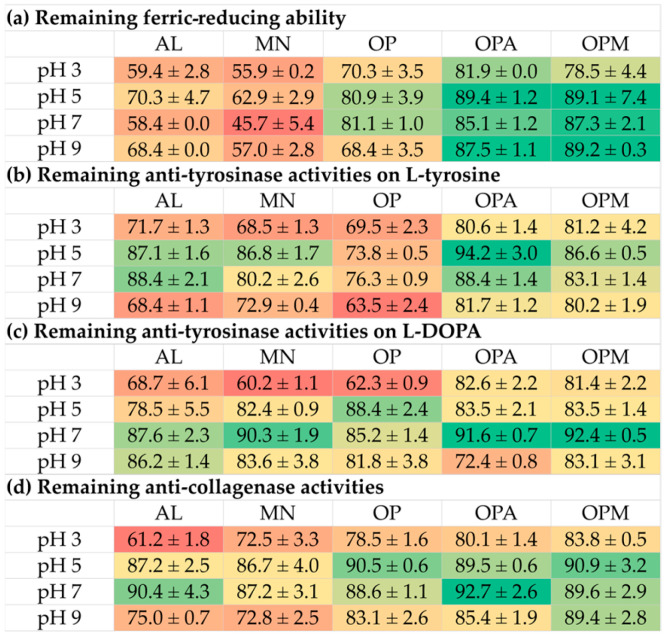
Heatmap presenting the effects of pH on stability, expressed as remaining biological activities: (**a**) ferric-reducing ability; (**b**) anti-tyrosinase activities on L-tyrosine; (**c**) anti-tyrosinase activities on L-DOPA; (**d**) anti-collagenase activities of allulose (AL), mannose (MN), *G. max* oligopeptide (OP), *G. max* oligopeptide-allulose (OPA), and *G. max* oligopeptide-mannose (OPM) after storage in various pH of 3, 5, 7, and 9 for 4 weeks. The color gradient represents the data values, where green indicates the highest values and red represents the lowest values. Intermediate values are shown in shades transitioning from green to yellow to red.

The effects of pH are presented in Figure 6. The decline in biological activities was observed over the storage period. Ferric-reducing abilities were found to decline under all pH conditions for the individual compounds, with monosaccharides experiencing a greater decline than oligopeptides, while the conjugates were found to be more stable. The heatmap as shown in Figure 7, would be more understandable as it presented the remaining activities after the storage for four weeks with a distinct color gradient. The findings highlighted that *G. max* oligopeptides combined with allulose (OPA) or mannose (OPM) exhibited superior stability compared to the individual components (AL, MN, OP). The OPA and OPM maintained high activity, especially at pH 5 to pH 9, while AL and MN showed significant reductions in all pH conditions.

Throughout the storage period, anti-tyrosinase activities on both L-tyrosine and L-DOPA substrates, along with anti-collagenase activities, exhibited a decline, particularly under acidic (pH 3) and alkaline (pH 9) conditions. Conversely, these activities remained more stable at neutral pH (pH 7). Notably, *G. max* oligopeptides with allulose (OPA) or mannose (OPM) demonstrated superior retention of anti-tyrosinase activity. In contrast, individual components (AL, MN, and OP) experienced significant activity reductions under both acidic and alkaline conditions. A similar pattern was observed for anti-collagenase activity. OPA and OPM maintained higher stability across all pH levels, whereas AL and MN showed substantial declines, especially at pH 3.

These findings suggested that the OP combinations with AL or MN effectively enhance the stability of bioactivities, making them promising candidates for applications requiring long-term stability in varying pH environments. This phenomenon can be attributed to the structural characteristics of these molecules. Generally, proteins and peptides have been known to face challenges in maintaining stability under pH variations [54]. This instability often leads to denaturation and a subsequent loss of biological functions [54]. This inherent instability poses significant limitations to the practical applications of proteins in various industrial and therapeutic contexts. Interestingly, the current study also revealed the instability of the monosaccharides, both AL and MN. The likely explanation could be attributed to conformational changes in the monosaccharide molecules. A previous study suggested that glucopyranose rings in glucose, maltose, sucrose, and amylose could undergo a conformational shift from C1 to 3B under alkaline conditions, impacting optical rotation due to the ionization of hydroxyl groups [55]. Since mannose is structurally similar to glucose (an epimer at C-2) and allulose can also form a pyranose ring, both may undergo similar conformational changes, potentially affecting their biological activities under alkaline conditions. In contrast, enhanced stability was observed in OPA and OPM, which may be attributed to the conjugation of *G. max* oligopeptides with AL or MN. This conjugation could confer greater resilience against pH-induced denaturation compared to the individual components. The findings were in line with Wijaya et al. (2017), who reported that covalent complexes or conjugates of proteins and polysaccharides were stable and not responsive to changes in pH or ionic strength, in contrast to non-covalent complexes [56]. The conjugation of proteins and polysaccharides has been suggested to improve protein stability and preserve their functional properties under processing conditions, including low pH or salt addition [56].

**Figure 8:**
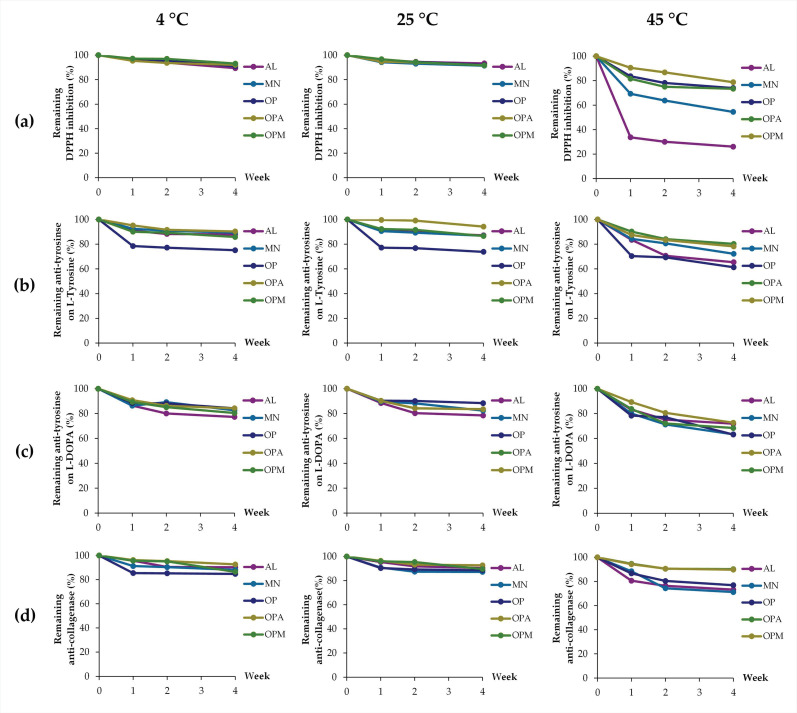
Effects of temperature on stability, expressed as remaining biological activities: (**a**) ferric-reducing ability; (**b**) anti-tyrosinase activities on L-tyrosine; (**c**) anti-tyrosinase activities on L-DOPA; (**d**) anti-collagenase activities of allulose (AL; purple), mannose (MN; cyan), *G. max* oligopeptide (OP; dark blue), *G. max* oligopeptide–allulose (OPA; yellow), and *G. max* oligopeptide–mannose (OPM; green) after storage in various temperature of 4 °C, 25 °C, and 45 °C, for up to 4 weeks.

**Figure 9:**
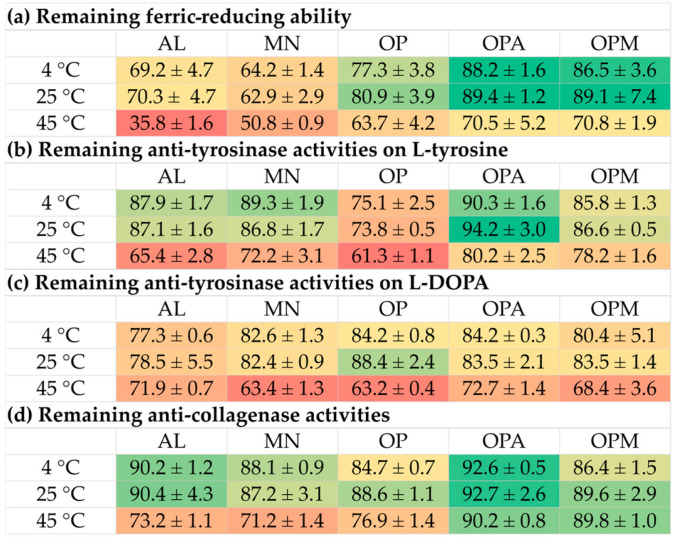
Heatmap presenting the effects of temperature on stability, expressed as remaining biological activities: (**a**) ferric-reducing ability; (**b**) anti-tyrosinase activities on L-tyrosine; (**c**) anti-tyrosinase activities on L-DOPA; (**d**) anti-collagenase activities of allulose (AL), mannose (MN), *G. max* oligopeptide (OP), *G. max* oligopeptide–allulose (OPA), and *G. max* oligopeptide–mannose (OPM) after storage in various temperature of 4 °C, 25 °C, and 45 °C for 4 weeks. The color gradient represents the data values, where green indicates the highest values and red represents the lowest values. Intermediate values are shown in shades transitioning from green to yellow to red.

Aside from pH, the effects of temperatures on the biological activities were also evaluated, as shown in Figure 8. The storage temperature significantly affected the remaining biological activities of all samples over four weeks. The heatmap in Figure 9 clearly displays a distinct color gradient, facilitating easier interpretation. At 4 °C and 25 °C, most samples retained high levels of DPPH radical scavenging, anti-tyrosinase, and anti-collagenase activities, with minimal degradation observed. However, at 45 °C, a marked reduction in all activities was evident, particularly in the AL and MN, which exhibited the lowest stability. AL and MN showed substantial reductions in activity, with the ferric reducing ability of AL decreasing sharply to 35.8 ± 1.6% at 45 °C. In contrast, OPA and OPM retained over 70% of their antioxidant capacity under the same condition. Similarly, anti-tyrosinase activities significantly decreased in AL and MN at 45 °C, while OPA and OPM maintained higher activity levels, indicating a protective effect of the *G. max* oligopeptide–monosaccharide conjugates. Notably, anti-collagenase activity was the most stable, with OPA and OPM preserving over 89% of activity even at 45 °C.

Therefore, it can be noted that the *G. max* oligopeptide–monosaccharide conjugates exhibited superior stability across all tested activities under elevated temperature conditions. These findings suggest that the conjugates, particularly the OPM complex, effectively enhanced the thermal stability of bioactive compounds, making them suitable for use in cosmeceutical or nutraceutical products requiring long-term storage under varying temperatures. The results align with previous studies indicating that combining proteins and polysaccharides through non-enzymatic browning, such as the Maillard reaction, can enhance heat stability [57]. Zhao et al. (2021) reported that glycosylated proteins of soy hull hemicellulose-soy protein isolate produced via the Maillard process have demonstrated improved thermal stability compared to the individual biopolymers [58]. The combination of proteins and polysaccharides in food systems exerts a synergistic effect, markedly improving the stability of dispersed phases beyond what either component can achieve individually [56]. Wijaya et al. (2017) also suggested that conjugations or covalent bonding between proteins and polysaccharides improves protein stability and preserves their functional properties under processing conditions, including heat treatment [56]. Not only did conjugation with polysaccharides improve protein stability but the stability of polysaccharides was also enhanced through conjugation with proteins. According to Smith et al. (2022), chemical cleavage of carbohydrate bonds in the polysaccharide reduced vaccine immunogenicity; however, sufficient stability could be attained through polysaccharide-protein conjugation [59].

**Figure 10:**
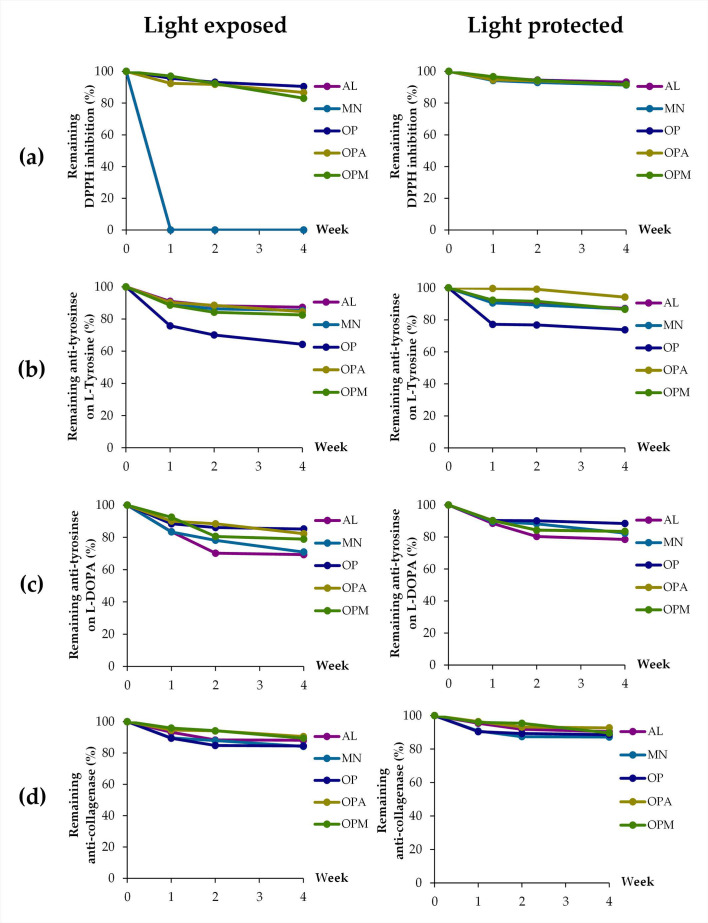
Effects of light exposure on stability, expressed as remaining biological activities: (**a**) ferric-reducing ability; (**b**) anti-tyrosinase activities on L-tyrosine; (**c**) anti-tyrosinase activities on L-DOPA; (**d**) anti-collagenase activities of allulose (AL; purple), mannose (MN; cyan), *G. max* oligopeptide (OP; dark blue), *G. max* oligopeptide–allulose (OPA; yellow), and *G. max* oligopeptide–mannose (OPM; green) after storage in various temperature of 4 °C, 25 °C, and 45 °C, for up to 4 weeks.

**Figure 11:**
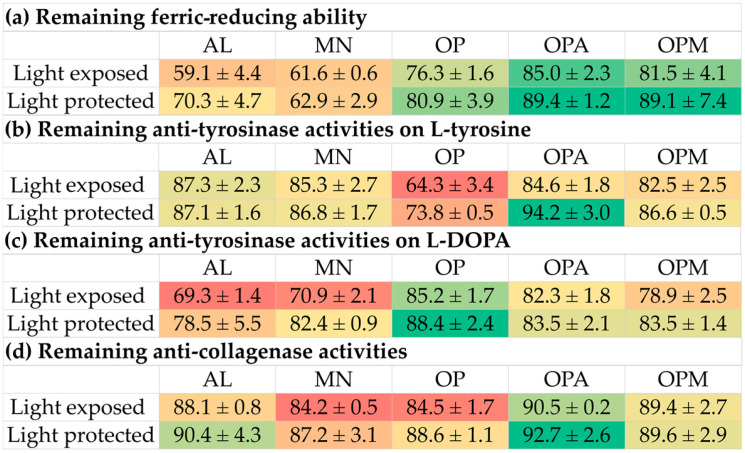
Heatmap presenting the effects of light exposure on stability, expressed as remaining biological activities: (**a**) ferric-reducing ability; (**b**) anti-tyrosinase activities on L-tyrosine; (**c**) anti-tyrosinase activities on L-DOPA; (**d**) anti-collagenase activities of allulose (AL), mannose (MN), *G. max* oligopeptide (OP), *G. max* oligopeptide–allulose (OPA), and *G. max* oligopeptide–mannose (OPM) after storage in various temperature of 4 °C, 25 °C, and 45 °C for 4 weeks. The color gradient represents the data values, where green indicates the highest values and red represents the lowest values. Intermediate values are shown in shades transitioning from green to yellow to red.

The effect of light exposure on biological activities was also evaluated. The results as shown in Figure 10 and the heatmap in Figure 11 demonstrated that light exposure significantly impacted the stability of biological activities, particularly antioxidant and anti-tyrosinase activities. Among the samples, MN showed the most rapid degradation, especially in DPPH radical scavenging, where its activity dropped sharply within the first week. In contrast, the *G. max* oligopeptide–monosaccharide conjugates, both the OPA and OPM, exhibited superior stability, retaining higher levels of activity (above 80%) under both light-exposed and light-protected conditions. Light protection notably preserved the activities of all samples, with minimal reductions observed over four weeks, particularly in OPA and OPM. Similarly, anti-tyrosinase activities on L-tyrosine and L-DOPA substrates were better retained in the light-protected conditions, with OPA showing the highest stability (94.2 ± 3.0% and 88.4 ± 2.4%, respectively). The OP exhibited the most degradation in anti-tyrosinase activities under light exposure. Interestingly, anti-collagenase activity was the least affected by light exposure, showing only slight decreases throughout the storage period. These findings suggested that the conjugation process effectively enhanced the thermal and photo-stability of the bioactive compounds, making OPA and OPM promising candidates for use in cosmeceutical applications requiring long-term stability under environmental stress.

Overall, the current study highlighted the improved bioactivities of the oligopeptide–monosaccharide conjugates under various pH levels, thermal conditions, and light exposure. These findings highlighted the potential of glycation via the Maillard reaction as a promising strategy to improve the functional and physicochemical properties of peptide-based compounds [60,61]. However, comprehensive, in-depth evaluations of the chemical composition, particularly the identification and characterization of metabolites formed under varying conditions, would be essential to gain a more complete understanding of their stability and bioactivity. Additional analytical techniques such as high-performance liquid chromatography coupled with tandem mass spectrometry (HPLC–MS/MS) or nuclear magnetic resonance (NMR) spectroscopy would be recommended for the identification and structural elucidation of metabolites formed under various conditions, thereby providing deeper insights into the chemical composition and potential bioactivity of the oligopeptide–monosaccharide conjugates.

## 4. Conclusions

*G. max* oligopeptide–monosaccharide conjugates, both OPA and OPM, demonstrated strong potential as multifunctional active ingredients for cosmetic and cosmeceutical applications due to their superior bioactivities and enhanced stability. OPA exhibited the strongest antioxidant capacity, while OPM was most effective in inhibiting tyrosinase, making it promising for skin-whitening formulations. Both OPA and OPM also significantly enhanced collagenase inhibition, supporting their anti-aging potential. Additionally, oligopeptide–monosaccharide conjugation not only improved bioactivity but also enhanced pH, thermal, and light stability, making these conjugates promising candidates for developing cosmeceutical and nutraceutical products with skin-brightening, anti-aging, and antioxidant properties suitable for long-term storage and effective use under environmental stress. However, to gain a more comprehensive understanding of the stability and mechanism of action of these conjugates, further investigations focusing on their detailed chemical composition and the metabolites formed during instability events are necessary. Additionally, further research focusing on their safety and efficacy in human clinical trials would provide more evidence to support their commercial applications.

## Figures and Tables

**Table 1:** Anti-tyrosinase activities of *G. max* oligopeptide–monosaccharide conjugates.

Samples	Anti-Tyrosinase Activities (%)	
	L-Tyrosine	L-DOPA
KA	98.0 ± 0.4 ^a^	72.6 ± 0.5 ^a^
AL	48.7 ± 2.3 ^b^	19.8 ± 1.4 ^e^
MN	32.2 ± 6.4 ^c^	17.2 ± 0.5 ^e^
OP	7.6 ± 3.2 ^d^	26.2 ± 1.4 ^d^
OPA	44.4 ± 3.2 ^b^	31.5 ± 1.9 ^c^
OPM	39.9 ± 3.3 ^b^	44.4 ± 1.7 ^b^

Note: KA = kojic acid; AL = allulose; MN = mannose; OP = *G. max* oligopeptide; OPA = *G. max* oligopeptide–allulose; OPM = *G. max* oligopeptide–mannose. The final concentration of the tested sample was 0.5 mg/mL. Different letters denote significant differences in the anti-tyrosinase activities among samples, as determined by ANOVA followed by Tukey’s test (*p* < 0.05).

**Table 2:** Irritation potentials of *G. max* oligopeptide–monosaccharide conjugates.

Samples	Irritation Score (IS)	Irritation Severity
1% *w*/*v* SLS	12.5 ± 0.3 ^a^	Severe irritation
NSS	0.0 ± 0.0 ^b^	No irritation
AL	0.0 ± 0.0 ^b^	No irritation
MN	0.0 ± 0.0 ^b^	No irritation
OP	0.0 ± 0.0 ^b^	No irritation
OPA	0.0 ± 0.0 ^b^	No irritation
OPM	0.0 ± 0.0 ^b^	No irritation

Note: Sodium lauryl sulfate (SLS) at 1% *w*/*v* served as a positive control, while normal saline solution (NSS) served as a negative control. The tested sample of allulose (AL), mannose (MN), *G. max* oligopeptide (OP), *G. max* oligopeptide–allulose (OPA), and *G. max* oligopeptide–mannose (OPM) were tested at the concentration of 1% *w*/*w*. Different letters denote significant differences in the irritation score among samples, as determined by ANOVA followed by Tukey’s test (*p* < 0.05).

## Data Availability

The original contributions presented in the study are included in the article; further inquiries can be directed to the corresponding author.

## Abbreviations

The following abbreviations are used in this manuscript:

AL	allulose
DPPH	2,2-Diphenyl-1-picrylhydrazyl
HET-CAM	hen’s egg chorioallantoic membrane assay
IS	irritation score
MN	mannose
OP	*G. max* oligopeptide
OPA	*G. max* oligopeptide–allulose conjugate
OPM	*G. max* oligopeptide–mannose conjugate
